# Impact of Gastrojejunostomy Configuration on Delayed Gastric Emptying Following Pancreaticoduodenectomy: A Single-Surgeon Retrospective Study

**DOI:** 10.3390/life15101521

**Published:** 2025-09-26

**Authors:** Forat Swaid, Muhammad Masalha, Rajaa Elias, Ahmed Asadi, Osama Knaaneh, Monther Graieb, Miguel Gorenberg, Mohammad Sheikh-Ahmad, Walid Shalata, Abed Agbarya

**Affiliations:** 1 Department of Surgery, Tzafon Medical Center, Poriya 1528001, Israel; 2 Department of Surgery, Bnai-Zion Medical Center, 47 Eliyahu Golomb Avenue, Haifa 3339419, Israel; 3 The Ruth and Bruce Rappaport Faculty of Medicine, Technion, Israel Institute of Technology, 1 Efron Street, Haifa 3339101, Israel; 4 Department of Nuclear Medicine, Bnai Zion Medical Center, Haifa 3339419, Israel; 5 Institute of Endocrinology, Bnai Zion Medical Center, 47 Eliyahu Golomb Avenue, Haifa 3339419, Israel; 6 The Legacy Heritage Cancer Center, Dr. Larry Norton Institute, Soroka Medical Center, Beer Sheva 8410501, Israel; 7 Faculty of Health Sciences, Ben Gurion University of the Negev, Beer Sheva 8410501, Israel; 8 Institute of Oncology, Bnai-Zion Medical Center, 47 Eliyahu Golomb Avenue, Haifa 3339419, Israel

**Keywords:** pancreaticoduodenectomy, delayed gastric emptying, gastrojejunostomy, antecolic, transmesocolic, surgical complications, reconstruction technique, pancreatic surgery

## Abstract

Delayed gastric emptying (DGE) is a significant complication following pancreaticoduodenectomy, affecting 20–40% of patients and impacting hospital stay, healthcare costs, and adjuvant therapy initiation. Different gastrojejunostomy configurations have been investigated to reduce DGE rates, with conflicting results presented in the literature. This retrospective study analyzed 65 consecutive patients who underwent pylorus-preserving pancreaticoduodenectomy at Bnai-Zion Medical Center between August 2018 and December 2023. All procedures were performed by a single experienced surgeon using either antecolic (AC, *n* = 25) or transmesocolic (TMC, *n* = 40) reconstruction. DGE was classified according to International Study Group of Pancreatic Surgery criteria. The statistical analysis included logistic regression to identify risk factors. The overall DGE incidence was 23.1% (15/65 patients). The AC group demonstrated significantly higher DGE rates compared to the TMC group (40% vs. 12.5%, *p* = 0.01). Logistic regression analysis revealed a 5.91-fold increased risk of DGE with AC reconstruction (OR: 5.91; 95% CI: [1.44, 24.25]; *p* = 0.014). All severe DGE cases (grades B and C) occurred exclusively in the AC group. Hospital stay was significantly longer in DGE patients (median: 26 vs. 13 days, *p* < 0.001). Other variables, including age, gender, smoking, diabetes, BMI, and surgical approach, showed no significant association with DGE. In this single-surgeon study, antecolic reconstruction was associated with significantly higher rates and severities of delayed gastric emptying compared to transmesocolic reconstruction. These findings suggest that reconstruction technique represents a modifiable risk factor for DGE prevention, though larger prospective studies are needed to confirm these results.

## 1. Introduction

Delayed gastric emptying (DGE) represents one of the most prevalent complications following pancreaticoduodenectomy (PD), with reported incidence rates ranging from 20% to 40% across various studies [1,2,3,4,5]. While generally not life-threatening, DGE significantly impacts patient outcomes through prolonged hospitalization, increased healthcare costs, elevated risk of aspiration pneumonia, higher readmission rates, and potential delays in initiating adjuvant chemotherapy when clinically indicated [1,2,3,4,5,6].

The International Study Group of Pancreatic Surgery (ISGPS) has established standardized criteria for defining and classifying DGE into three grades based on clinical severity and management requirements [7]. Grade A DGE typically responds to conservative management including nasogastric decompression, parenteral nutrition, and prokinetic medications [5,6]. However, patients with higher-grade DGE (grades B and C) may require endoscopic interventions or surgical revision [8].

Multiple pathophysiological mechanisms have been implicated in DGE development following pancreaticoduodenectomy. These include vagal nerve disruption during surgical dissection, gastroduodenal ischemia, altered gastrointestinal hormone regulation due to duodenectomy, and impaired gastric motility mechanisms [5,6]. Additionally, other major postoperative complications, particularly postoperative pancreatic fistula (POPF) and intra-abdominal fluid collection, have been associated with increased DGE risk [1,2,3,9].

Given the significant clinical impact of DGE, considerable research has focused on identifying preventive strategies and modifiable risk factors. Among these interventions, the gastrojejunostomy (GJ) anastomotic configuration has received particular attention [9]. Specifically, investigators have extensively studied whether the method of bringing the jejunal loop to the stomach—either via an antecolic (AC) or a transmesocolic (TMC) approach—influences DGE incidence [1]. However, results from published studies and meta-analyses remain inconsistent and contradictory [5,8].

A significant limitation of previous investigations is their heterogeneous nature, often involving multiple institutions, surgeons, and varying definitions of DGE, postoperative care protocols, and surgical techniques. Such heterogeneity makes it challenging to draw definitive conclusions regarding the optimal reconstruction approach.

This retrospective study presents a consecutive series of pancreaticoduodenectomies performed exclusively by a single experienced surgeon, thereby ensuring consistency in definitions, protocols, and surgical techniques for both antecolic and transmesocolic gastrojejunostomy construction. To our knowledge, this represents the first report examining the impact of gastrojejunostomy configuration on DGE incidence across open, laparoscopic, and robotic pancreaticoduodenectomy approaches within a single-surgeon experience.

The primary objective of this study was to determine DGE prevalence using standardized ISGPS criteria and compare the impact of antecolic versus transmesocolic reconstruction on DGE incidence and severity following pancreaticoduodenectomy.

## 2. Materials and Methods

### 2.1. Study Design and Ethical Considerations

This retrospective observational cohort study was conducted at Bnai-Zion Medical Center following approval by the Institutional Review Board in accordance with the Declaration of Helsinki (protocol number 0019-24-BNZ). The study analyzed all consecutive patients who underwent pylorus-preserving pancreaticoduodenectomy (PPPD), or Whipple or total pancreatectomy (TP), with either antecolic or transmesocolic reconstruction between August 2018 and December 2023. Given the retrospective nature of the study, the requirement for individual patient consent was waived by the ethics committee.

### 2.2. Patient Population and Inclusion Criteria

The study included 65 consecutive patients who underwent pancreaticoduodenectomy during the study period. Of the 65 resections in this study, 5 were total pancreatectomies and 60 were pancreatoduodenectomies (PD). Among the 60 pancreaticoduodenectomy cases, 45 were pylorus-preserving (PPPD) and 15 were classic Whipple procedures. All patients had various surgical indications, including pancreatic adenocarcinoma, periampullary tumors, periampullary carcinoma, and pancreatic cyst. The cohort included 48 patients with pancreatic ductal adenocarcinoma (AJCC stage I–III), 16 with periampullary carcinoma, and 1 with other diagnoses. Neoadjuvant chemotherapy was administered in 6 patients (9.2%), as shown in Table 1. Patients were categorized into two groups based on the reconstruction technique employed: antecolic reconstruction (*n* = 25, 38.5%) or transmesocolic reconstruction (*n* = 40, 61.5%). All TP patients had a gastrojejunostomy constructed via the transmesocolic route in our study.

### 2.3. Data Collection

Medical records were systematically reviewed for comprehensive data collection spanning preoperative, intraoperative, and postoperative variables. Preoperative data included patient demographics (age and gender), comorbidities (diabetes mellitus and smoking history), and nutritional status (body mass index and serum albumin levels). Intraoperative variables encompassed surgical approach (laparoscopic, open, or robotic-assisted), type of pancreatic reconstruction, and extent of lymphadenectomy. Postoperative outcomes included DGE occurrence and grading according to ISGPS criteria, hospital length of stay, complications such as small bowel obstruction, and surgical margin status.

### 2.4. Surgical Technique

All procedures were performed by a single experienced pancreatic surgeon with expertise in both antecolic and transmesocolic reconstruction techniques. The surgical approach varied based on patient anatomy characteristics, tumor location, and surgeon preference (open, laparoscopic, and robotic-assisted surgery techniques). Standard pancreaticoduodenectomy or pylorus-preserving pancreaticoduodenectomy was performed according to established principles, with systematic lymphadenectomy performed in all cases.

Reconstruction Techniques:

- Antecolic (AC) Reconstruction: The jejunal loop was brought anterior to the transverse colon for gastrojejunostomy anastomosis.
- Transmesocolic (TMC) Reconstruction: The jejunal loop was passed through a defect created in the transverse mesocolon for posterior anastomosis.

The gastrojejunostomy was fashioned end-to-side, using a two-layer hand-sewing technique in all cases (regardless of AC or TMC configuration).

The reconstruction route (AC vs. TMC) was selected based on intraoperative considerations: in open surgery cases a retrocolic (TMC) route was usually utilized, whereas in laparoscopic/robotic surgery cases an antecolic route was generally favored for technical facility.

No patient-specific anatomical factors necessitated one configuration; the choice reflected surgeon preference and the surgical approach being used.

No prophylactic nasojejunal feeding tubes were placed. Nasogastric tubes were removed early (within 24–48 h post-op) to encourage return of gastric function, unless the patient developed DGE requiring prolonged decompression.

### 2.5. Delayed Gastric Emptying Definition and Classification

DGE was defined and classified according to the standardized ISGPS criteria [7]. Grade A DGE was characterized by the requirement for nasogastric decompression for 4–7 days or an inability to tolerate solid oral intake by postoperative day 7. Grade B DGE required nasogastric decompression for 8–14 days or an inability to tolerate solid food by postoperative day 14. Grade C DGE was defined as the need for nasogastric decompression for more than 14 days or an inability to tolerate solid food by postoperative day 21, often requiring interventional procedures.

### 2.6. Statistical Analysis

Statistical analysis was performed using SPSS version 28.0 (IBM Corporation, Armonk, NY, USA). Descriptive statistics were calculated as means with standard deviations for normally distributed continuous variables, medians with interquartile ranges for non-normally distributed variables, and frequencies with percentages for categorical variables. Distribution normality was assessed using the Kolmogorov–Smirnov test.

Continuous variables were compared between groups using Student’s *t*-test for normally distributed data or the Mann–Whitney U test for non-normally distributed data. Categorical variables were analyzed using Pearson’s chi-square test or Fisher’s exact test as appropriate.

Univariate logistic regression analysis was performed to identify potential risk factors for DGE development. Variables with clinical significance were included in the analysis regardless of *p*-value. Odds ratios (ORs) with 95% confidence intervals (CIs) were calculated. Statistical significance was defined as *p* < 0.05.

## 3. Results

### 3.1. Patient Demographics and Baseline Characteristics

The study cohort comprised 65 patients with a mean age of 68.8 ± 12.6 years. Male patients accounted for 54% (35/65) of the cohort. Baseline demographic and clinical characteristics were generally comparable between the antecolic (AC) and transmesocolic (TMC) reconstruction groups (Table 1).

The mean age was similar between groups (AC: 69.4 ± 14.3 years vs. TMC: 68.5 ± 11.6 years; *p* = 0.78). Body mass index values were also comparable (AC: 24.8 ± 4.2 kg/m^2^ vs. TMC: 25.2 ± 3.8 kg/m^2^; *p* = 0.76). The prevalence of diabetes mellitus was numerically higher in the TMC group, though not statistically significant (47.5% vs. 28%; *p* = 0.12).

Notable baseline differences: A statistically significant difference was observed in mean preoperative serum albumin levels, with the AC group demonstrating lower values compared to the TMC group (3.4 ± 0.53 g/dL vs. 3.8 ± 0.56 g/dL; *p* = 0.013). Additionally, the distribution of surgical approaches differed significantly between groups (*p* < 0.001), with the AC group undergoing more laparoscopic procedures (48% vs. 5%), while the TMC group had a higher proportion of open surgical procedures (75% vs. 36%).

Post-surgery, there were five cases of clinical leak manifestation; two were in the AC group and three in the TMC group. All leak cases were of mild grade and treated by a conservative approach. Postoperative pancreatic fistula was reported in four cases; one was in the AC group and three in TMC cohort. Two of the pancreatic fistula cases were graded mild to severe and needed reoperation. The other two were of mild grade and conservatively treated. Hospital stay for the group of patients with complications was in the range of 10–32 days, and the median was 16 days.

### 3.2. Delayed Gastric Emptying Incidence and Severity

The overall incidence of delayed gastric emptying in the entire cohort was 23.1% (15/65 patients) (Table 2). The antecolic reconstruction group demonstrated a significantly higher DGE rate compared to the transmesocolic group (40.0% [10/25] vs. 12.5% [5/40], respectively; *p* = 0.01) (Table 3). The analysis of DGE incidence and key outcomes stratified by procedure type (PPPD vs. Whipple vs. TP) revealed that DGE was observed in PPPD patients more frequently than in classic Whipple patients in our series (PPPD DGE rate: 73.3% (11/15; seven in AC cohort; four in TMC cohort); Whipple DGE rate: 26.7% (4/15; three in AC cohort, one in TMC cohort)), consistent with the notion that preserving the pylorus can increase DGE risk [10]. All total pancreatectomy patients (*n* = 5) are also reported in this table; none of the TP patients in our cohort developed DGE. However, due to the small sample size in the Whipple and TP subgroups, these differences did not reach statistical significance.

DGE Severity Analysis: Among the 15 patients who developed DGE, the majority were classified as grade A (12 patients, 80%), while 3 patients (20%) experienced severe DGE classified as grades B and C according to the ISGPS criteria. Notably, all three cases of severe DGE (grades B and C) occurred exclusively in the antecolic reconstruction group, representing 30% (3/10) of DGE cases in this group compared to 0% in the TMC group.

### 3.3. Clinical Impact and Associated Outcomes

Patients who developed DGE experienced significantly prolonged hospitalization compared to those without DGE. The median hospital stay was 26 days (interquartile range (IQR): 19–46 days) for patients with DGE versus 13 days (IQR: 10–22 days) for those without DGE (*p* < 0.001) (Table 2).

Small bowel obstruction occurred more frequently in patients with DGE compared to those without (20% [3/15] vs. 2% [1/50]; *p* = 0.037), suggesting a potential association between these complications.

### 3.4. Risk Factor Analysis

Logistic regression analysis confirmed that reconstruction technique was the primary factor associated with DGE development. Antecolic reconstruction independently increased the risk of DGE compared to transmesocolic reconstruction, with an odds ratio of 5.91 (95% CI: 1.44–24.25; *p* = 0.014) (Table 3).

Other potential risk factors analyzed, including patient age, gender, smoking status, diabetes mellitus, body mass index, preoperative albumin levels, surgical approach (laparoscopic, open, or robotic), extent of lymphadenectomy, and lymph node involvement, did not demonstrate statistically significant associations with DGE development.

## 4. Discussion

This single-surgeon retrospective study demonstrates a significant association between gastrojejunostomy reconstruction technique and delayed gastric emptying following pancreaticoduodenectomy. Our findings indicate that antecolic reconstruction carries a nearly six-fold increased risk of DGE compared to transmesocolic reconstruction, with all severe DGE cases occurring exclusively in the antecolic group. Of note, the total pancreatectomy patients who were included in the study were without DGE, despite their physiological differences (e.g., complete loss of pancreatic and duodenal function) that could affect gastric emptying. No clear impact was observed for this limited subset of surgery type; hence, the conclusions pertain primarily to the standard PD cases of the current study. The present data, indicating a higher DGE rate following PPPD compared to classic Whipple resection, aligns with prior reports that pylorus-preserving PD may predispose patients to DGE [10]. However, our sample size for the Whipple subgroup was limited (*n* = 15), precluding definitive conclusions.

The standardized anastomosis technique and early NG tube removal used in the present study were consistent across all cases; therefore, differences in DGE are less likely to be due to variations in technique or tube management.

### 4.1. Comparison with Existing Literature

The overall DGE incidence of 23.1% in our cohort falls within the commonly reported range of 20–40% for pancreaticoduodenectomy procedures [1,2,3,5,11,12,13,14,15]. However, our findings contrast with several published studies regarding the optimal reconstruction technique.

Several large studies have reported favorable outcomes with antecolic reconstruction. Sahora et al. analyzed 800 patients and found lower DGE rates with antecolic compared to retrocolic reconstruction (15% vs. 21%, respectively) [1]. Similarly, studies by Varghese et al. [13] and Kim et al. [6] demonstrated decreased DGE risk with antecolic gastrojejunostomy. Kurosaki observed that antecolic duodenojejunostomy following PPPD reduced DGE incidence [16]. A prospective trial by Tani et al. showed that antecolic duodenojejunostomy during PPPD decreased DGE incidence in comparison to retrocolic [17]. These findings are contrary to our results and highlight the ongoing debate in the literature.

Conversely, our results align with recent studies that have questioned the superiority of antecolic reconstruction. Geng et al. reported an increased risk of DGE with antecolic reconstruction compared to the transmesocolic approach (odds ratio: 1.51; 95% CI: [1.07, 2.15]) [8], which is consistent with our findings despite the lower magnitude of effect.

Robinson et al. reported that patients with DGE had significantly longer hospital stays than non-DGE patients [18], which is line with the current study findings indicating that SBO prevalence in the DGE group was statistically significantly different from that in the non-DGE group. Brown et al.’s study of surgical complications after pancreatic duodenectomy found that the incidence of SBO was 4.3% [19].

In the current study diabetes mellitus (DM) prevalence was not statistically different among DGE and non DGE patients. These results are in line with Robinson et al. report who found no difference between DGE and no-DGE groups [18]. Nevertheless, Qu et al. showed that preoperative diabetes was a predictive risk factor for DGE [20].

The present study cohorts of patients with DGE or without DGE had similar BMI 25 kg/m^2^ mean ± SD. However, Robinson et al. studied peri-operative risk factors related to DGE in patients who underwent pancreaticoduodenectomy [18]. A higher rate of patients with BMI ≥ 35 was found among those experiencing DGE vs. those without DGE (15% vs. 9%, respectively).

Varghese et al.’s review analysis of randomized trials emphasized that postoperative pancreatic fistula is a risk factor for DGE after PD [13]. Robinson et al.’s study as well as Mohammed et al.’s study also found that DGE patients had an increased incidence of postoperative pancreatic fistula POPF [15,18].

Lymphadenectomy performed on patients taking part in the current study was of a similar extent both for the DGE group and non-DGE patients. Pancreatic cancer patients might have up to 30% lymph node disease that needs to be dissected to prevent infiltration of malignant cells [21].

The present study showed that both cohorts of patients with and without DGE had a comparable mean age, and similar findings were recorded for the antecolic and transmesocolic groups. Dai et al.’s review of clinical risk factors of DGE following PPPD or PD found that older age was associated with an increased incidence or DGE [22]. Degisors et al. found 10% DGE incidence among patients who had undergone distal pancreatectomy. The predisposing factors were an age > 75 years and an open surgical approach. Patients experiencing DGE had prolonged hospital stays [23]. The study results were unlike those of the current research, which could be due to the different surgery conditions and different patient populations.

Several research studies aiming to investigate possible factors that contribute to a decrease in DGE were published. A report by Manes et al. suggested that antecolic reconstruction with pylorus dilatation could lower the rate of DGE following PPPD [24]. Lee et al. have shown that pylorus-resecting pancreaticoduodenectomy reduced the incidence of DGE [25]. Kurahara et al. concluded that subtotal stomach-preserving pancreaticoduodenectomy was associated with lower DGE prevalence than occurred with PPPD [26]. Other risk factors, such as smoking, pancreatic fistula, and bilirubin levels > 6 mg/dL, were considered to be related to DGE syndrome [27]. Murakami et al.’s [28] findings indicated that pancreatic fibrosis following PPPD was associated with 13% DGE.

Multiple randomized controlled trials and meta-analyses have reported no significant differences between reconstruction techniques. Joliat et al. conducted a meta-analysis of six randomized controlled trials and found no differences between antecolic and retrocolic reconstruction in terms of DGE rates, hospital stay, or DGE severity grades [5]. Similarly, a multicenter RCT by Toyama et al., and a Cochrane review by Hüttner et al., concluded that there was little to no difference in DGE rates between the two techniques [29,30].

### 4.2. Potential Mechanisms and Clinical Implications

The higher incidence and severity of DGE observed with antecolic reconstruction in our study may be attributed to anatomical and physiological factors. While antecolic reconstruction theoretically provides a more direct path for gastric contents, the anterior positioning may subject the anastomosis to greater external compression from the overlying structures or increased mobility that could compromise optimal function.

In contrast, transmesocolic reconstruction positions the jejunal limb more posteriorly with potential stabilization through suturing to the mesocolic defect, which may reduce anastomotic tension and minimize the risk of kinking or obstruction [31,32]. This anatomical advantage might explain the lower DGE rates observed in our TMC group.

Few studies use DGE classification according to ISGPS subgroup grades. Sahora et al. reported 9% grade A DGE for their antecolic group, which was significantly different from that in the RC group, while grades B and C were of similar incidence [1]. Imamura et al. found that DGE grades B/C had lower incidence in AC than in RC patients [33]. In addition, Joliat et al. found no statistically significant difference in distinct DGE grades A, B, and C between patients who had undergone different types of reconstruction [5].

### 4.3. Study Strengths and Clinical Significance

The primary strength of this investigation lies in its single-surgeon design, which eliminates the technical and procedural variability inherent in multi-surgeon or multi-institutional studies. This consistency allows for a more accurate assessment of the specific impact of reconstruction technique on DGE incidence while minimizing confounding variables related to surgical technique variations. All surgeries were performed by a single experienced pancreatic surgeon, reducing variability. By the time of this series, the surgeon’s technique had matured, minimizing the influence of a learning curve on outcomes. The study period did coincide with the introduction of minimally invasive PD at our center; however, even the initial laparoscopic cases were proctored and approached after significant experience with open surgery, which we believe mitigated drastic learning-curve effects on DGE. Moreover, the single-surgeon nature of the study is a strength that ensures technical consistency. There was no clear temporal trend of decreasing DGE in later cases—in fact, DGE was observed more frequently in the later period when AC was commonly used (reflecting the technique difference rather than general skill improvement). This paradox is mentioned here to argue that the higher DGE with AC is unlikely to be due to early experience (since AC was predominantly utilized after the surgeon had gained extensive experience).

Additionally, the standardized use of ISGPS criteria for DGE definition and grading enhances the reliability and reproducibility of our results, facilitating meaningful comparisons with other studies in the literature.

The finding that all severe DGE cases (grades B and C) occurred exclusively in the antecolic group provides important clinical insight, suggesting that reconstruction technique may influence not only the incidence but also the severity of this complication.

Our study adds value by providing additional evidence (albeit from a smaller cohort) in a controlled setting, and by including under-represented scenarios (minimally invasive PDs and total pancreatectomies) to enrich the understanding of how GJ configuration might influence DGE.

### 4.4. Study Limitations

Several limitations must be acknowledged in interpreting these results. The retrospective design of this study inherently limits the ability to control for all potential confounding variables. The relatively small sample size, while adequate for statistical analysis, may limit the generalizability of our findings to broader populations.

A significant baseline difference in preoperative albumin levels was observed between groups, with the antecolic group having lower levels. This difference may represent an important confounding factor, as nutritional status can influence postoperative outcomes, including gastric motility recovery [34]. However, no statistical difference in albumin blood level was found between DGE patients and those without DGE. Of note 10/15 (67%) patients reporting DGE underwent AC (Table 1), while among patients without DGE, the AC group rate was 15/50 (30%) (Table 2). Although hypoalbuminemia can predispose patients to slower recovery, in our data, low albumin did not show a direct correlation with DGE occurrence, that the AC group’s higher DGE rate might partly reflect their poorer baseline nutritional status.

The non-randomized assignment of reconstruction techniques introduces potential selection bias, as the choice of technique may have been influenced by patient-specific factors, tumor characteristics, or temporal changes in surgeon preference during the study period. There was a temporal element: early in the series (when most cases were open surgery cases) TMC was more common, while later, as our minimally invasive program expanded, AC became more frequently used.

Furthermore, the significant difference in surgical approach distribution between groups (more laparoscopic procedures in the AC group) represents another potential confounding variable that could influence outcomes independently of reconstruction technique. The AC group’s higher DGE rate does not appear to be explained by the use of minimally invasive surgery (MIS), since our adjusted analysis found no difference in DGE between open surgery and MIS cases. Although, the non-random allocation of surgical approaches could introduce bias, it was mitigated through multivariate analysis.

### 4.5. Future Directions

Given the conflicting results in the literature and the limitations of retrospective studies, larger prospective randomized controlled trials are needed to definitively establish the optimal reconstruction technique for minimizing DGE risk. Such studies should standardize patient selection criteria, surgical techniques, and postoperative care protocols while ensuring adequate power to detect clinically meaningful differences.

Additionally, future research should investigate the underlying mechanisms responsible for the observed differences in DGE rates between reconstruction techniques, potentially through advanced imaging studies or physiological assessments of gastric emptying.

## 5. Conclusions

This single-surgeon retrospective study demonstrates that antecolic reconstruction was associated with significantly higher rates and severities of delayed gastric emptying compared to transmesocolic reconstruction following pancreaticoduodenectomy. The nearly six-fold increased risk of DGE with antecolic reconstruction, combined with the exclusive occurrence of severe DGE cases in this group, suggests that reconstruction technique represents a potentially modifiable risk factor for DGE prevention.

These findings have important clinical implications for surgical planning and may inform decision-making regarding the optimal reconstruction approach. However, given the conflicting literature and study limitations, larger prospective randomized trials are warranted to confirm these results and establish definitive evidence-based recommendations for gastrojejunostomy configuration in pancreaticoduodenectomy.

Clinical Relevance: Surgeons should consider these findings when planning reconstruction techniques while acknowledging that individual patient factors, institutional experience, and surgeon expertise remain important determinants in selecting the optimal approach for each case.

## Figures and Tables

**Table 1:** Baseline characteristics and outcomes by reconstruction technique.

Variable	Antecolic (*n* = 25)	Transmesocolic (*n* = 40)	Total (*n* = 65)	*p*-Value
Age (years), mean ± SD *	69.4 ± 14.3	68.5 ± 11.6	68.8 ± 12.6	0.78
Male gender, *n* (%)	17 (68)	18 (45)	35 (54)	0.07
Smoking, *n* (%)	6 (24)	10 (25)	16 (24.6)	0.93
DM *, *n* (%)	7 (28%)	19 (47.5)	26 (40)	0.12
BMI * (kg/m^2^), mean ± SD *	24.8 ± 4.2	25.2 ± 13.8 *(n* = 39)	25.1 ± 3.9	0.76
Albumin (g/dL), mean ± SD *	3.4 ± 0.53	3.8 ± 0.56	3.6 ± 0.57	0.013
Neoadjuvant chemotherapy treatment protocol, *n* (%)	3 (12)	3 (7.5)	6 (9.2)	*p* = 0.67
FLOT, *n* (%)	1 (4)	0	1 (1.5)	
FOLFIRNIOX, *n* (%)	2 (8)	3 (7.5)	5 (7.7)	
Surgical approach, *n* (%)				<0.001
Laparoscopic	12 (48)	2 (5)	14 (21.5)	
Open	9 (36)	30 (75)	39 (60)	
Robotic	4 (16)	8 (20)	12 (18.5)	
Surgery type, *n* (%)				
PPPD *, *n* (%)	14 (56)	31 (77.5)	45 (69.2)	
Whipple, *n* (5%)	9 (36)	6 (15)	15 (23.1)	
TP *, *n* (%)	2 (8)	3 (7.5)	5 (7.7)	
Surgical indication, *n* (%)				
Pancreatic adenocarcinoma, *n* (%)	17 (68)	31 (77.5)	48 (74)	
Periampullary tumor, *n* (%)	7 (28)	9 (22.5)	16 (24.5)	
Pancreatic cyst, *n* (%)	1 (4)	0	1 (1.5)	
Tumor staging, *n* (%)				
I	3 (12)	6 (15)	9 (13.85)	
II	18 (72)	29 (72.5)	47 (72.3)	
III	4 (16)	5 (12.5)	9 (13.85)	
Complication				
Leak, *n* (%)	2 (8)	3 (7.5)	5 (7.7)	
Pancreatic fistula, *n* (%)	2 (8)	2 (5)	4 (6.15)	
Hospital stay (days), median [IQR *]	16 [10–37]	15.5 [12–22]	16 [10–37]	1.00
DGE *, *n* (%)	10 (40)	5 (12.5)	15 (23.1)	0.01
DGE severity (grades B-C), *n* (%)	3/10 (30)	0/5 (0)	3/15 (20)	

* Abbreviations: BMI, body mass index; DGE, delayed gastric emptying; DM, diabetes mellitus; IQR, interquartile range; PPPD, pylorus-preserving pancreaticoduodenectomy; SD, standard deviation; TP, total pancreatectomy.

**Table 2:** Clinical characteristics by DGE status.

Variable	DGE * (*n* = 15)	No DGE (*n* = 50)	Total (*n* = 65)	*p*-Value
Age (years), mean ± SD *	70.2 ± 12.3	68.4 ± 12.8	68.8 ± 12.6	0.63
Male gender, *n* (%)	7 (47)	28 (56)	35 (54)	0.53
Smoking, *n* (%)	5 (33%)	11 (22%)	16 (24.6%)	0.37
DM *, *n* (%)	5 (33%)	21 (42%)	26 (40%)	0.55
BMI * (kg/m^2^), mean ± SD *	24.1 ± 3.5	25.3 ± 4 *(n* = 49)	25.0 ± 3.9	0.31
Albumin (g/dL), mean ± SD *	3.6 ± 0.56	3.8 ± 0.58	3.3 ± 0.57	0.86
Surgical approach, *n* (%)				0.47
Laparoscopic	4 (26.7)	10 (20)	14 (21.5)	
Open	7 (46.7)	32 (64)	39 (60)	
Robotic	4 (26.7)	8 (16)	12 (18.5)	
Hospital stay (days), median [IQR *]	26 [19–46]	13 [10–22]	16 [10–46]	<0.001
SBO *, *n* (%)	3 (20)	1(2)	4 (6.3)	0.037
Lymphadenectomy, mean ± SD *	12.9 ± 6.9	16.5 ± 6.8	15.7 ± 6.9	0.087
Negative resection margins, *n* (%)	12/14 (85.7)	46/49 (93.9)	58/63 (92.1)	0.31

* Abbreviations: BMI, body mass index; DM, diabetes mellitus; DGE, delayed gastric emptying; IQR, interquartile range; SBO, small bowl obstruction; SD, standard deviation.

**Table 3:** Reconstruction techniques and DGE prevalence.

Reconstruction Type	DGE * (*n* = 15)	No DGE (*n* = 50)	Total (*n* = 65)	DGE Rate	OR (95% CI)	*p*-Value
AC *, *n*	10	15	25 (38.5%)	10/40 40.0%	5.91 [1.44, 24.25]	0.014
TMC *, *n*	5	35	40 (61.5%)	5/40 12.5%		
Total	15	50	65	15/65 23%		

* Abbreviations: AC, antecolic; DGE, delayed gastric emptying; TMC, transmesocolic.

## Data Availability

The data presented in this study are available on request from the corresponding author due to privacy and ethical restrictions.

## Abbreviations

The following abbreviations are used in this manuscript:

AC	Antecolic reconstruction
AJCC	American Joint Committee on Cancer
BMI	Body mass index
CI	Confidence interval
DGE	Delayed gastric emptying
DM	Diabetes mellitus
GJ	Gastrojejunostomy
IQR	Interquartile range
ISGPS	International Study Group of Pancreatic Surgeons
MIS	Minimally invasive surgery
OR	Odds ratio
PD	Pancreaticoduodenectomy
PPPD	Pylorus-preserving pancreaticoduodenectomy
POPF	Post-operative pancreatic fistula
TMC	Trans-mesocolic reconstruction
TP	Total pancreatectomy
